# California Almond Yield Prediction at the Orchard Level With a Machine Learning Approach

**DOI:** 10.3389/fpls.2019.00809

**Published:** 2019-07-18

**Authors:** Zhou Zhang, Yufang Jin, Bin Chen, Patrick Brown

**Affiliations:** ^1^Department of Land, Air and Water Resources, University of California, Davis, Davis, CA, United States; ^2^Biological Systems Engineering, University of Wisconsin–Madison, Madison, WI, United States; ^3^Department of Plant Sciences, University of California, Davis, Davis, CA, United States

**Keywords:** almond orchard, nitrogen management, yield prediction, yield variation, central valley, remote sensing, machine learning

## Abstract

California’s almond growers face challenges with nitrogen management as new legislatively mandated nitrogen management strategies for almond have been implemented. These regulations require that growers apply nitrogen to meet, but not exceed, the annual N demand for crop and tree growth and nut production. To accurately predict seasonal nitrogen demand, therefore, growers need to estimate block-level almond yield early in the growing season so that timely N management decisions can be made. However, methods to predict almond yield are not currently available. To fill this gap, we have developed statistical models using the Stochastic Gradient Boosting, a machine learning approach, for early season yield projection and mid-season yield update over individual orchard blocks. We collected yield records of 185 orchards, dating back to 2005, from the major almond growers in the Central Valley of California. A large set of variables were extracted as predictors, including weather and orchard characteristics from remote sensing imagery. Our results showed that the predicted orchard-level yield agreed well with the independent yield records. For both the early season (March) and mid-season (June) predictions, a coefficient of determination (*R*^2^) of 0.71, and a ratio of performance to interquartile distance (RPIQ) of 2.6 were found on average. We also identified several key determinants of yield based on the modeling results. Almond yield increased dramatically with the orchard age until about 7 years old in general, and the higher long-term mean maximum temperature during April–June enhanced the yield in the southern orchards, while a larger amount of precipitation in March reduced the yield, especially in northern orchards. Remote sensing metrics such as annual maximum vegetation indices were also dominant variables for predicting the yield potential. While these results are promising, further refinement is needed; the availability of larger data sets and incorporation of additional variables and methodologies will be required for the model to be used as a fertilization decision support tool for growers. Our study has demonstrated the potential of automatic almond yield prediction to assist growers to manage N adaptively, comply with mandated requirements, and ensure industry sustainability.

## Introduction

World production of almond was 2.2 million tons in 2017, and the leading producers include United States, Australia, Spain, Iran and Italy (Food and Agriculture Organization of the United Nations [FAO], 2018). In the United States, almond production is concentrated in California, with a total value of annual production of over 5.3 billion dollars in 2015. California’s almond acreage has been rapidly growing, from 283,280 hectares in 2005 to 538,232 hectares in 2017 (National Agricultural Statistics Service [NASS], 2016). Due to its scale, nitrogen (N) management in almond has a large potential impact on groundwater quality. As many as 10% of public water-supply wells in California must be treated, since nitrate levels exceed the maximum contamination level (Harter, 2009). As a result, nitrogen management workplans^1^ are now required of almond growers statewide to meet the goal of reducing nitrogen losses to the environment. To optimize N management and ensure regulatory compliance, growers must apply N in accordance with predicted yield in each production unit, taking into account N available from all sources (fertilizer, composts and manures, irrigation water nitrogen). Yield prediction is therefore critical for N management. In addition, it can help growers make plans for the harvest, processing, and transport of the crop (Zarate-Valdez et al., 2015). Quantitative yield modeling is also needed to improve our understanding of how crop growth and yield respond to short term environmental stress, climate variability and long-term climate trends. Moreover, forecasting inter-year yield variation plays a key role in food security monitoring and market planning, and has the potential to help managing food production shocks (Iizumi et al., 2018). Accurately estimating crop yield, therefore, has broad implications for ecology, economics, and human society, e.g., through its impact on the optimal use of inputs (irrigation water, fertilizers) and other resources (machinery, labor) on the farm (Carletto et al., 2015; Hoffman et al., 2015).

Yield of an almond orchard is determined by a complex interplay of processes and varies significantly from year to year and from orchard to orchard. Statewide annual production, for example has varied from 791 kg/ha in 1986 to 2864 kg/ha in 2011 (California Agricultural Statistics Service [CASS], 2018). In California, commercial almond orchards are generally removed after 20–25 years average, and the almond tree does not bear significant fruit during the first 3–4 years after planting (Boriss and Brunke, 2005). Almond trees are moderately alternate bearing so that a higher yield in one year is often followed by a lower yield the following year (Boriss and Brunke, 2005). Almond is a deciduous tree and changes in seasonal weather patterns can alter the physiology of every growth stage, resulting in yield variation. For example, in the winter season from November to February, almond trees are dormant, followed by a period of warming in the spring which has a large influence on the subsequent flowering and yield potential (Luedeling, 2012). The almond production in California requires cross pollination by bees, and yield is thus dependent on the presence of two compatible almond cultivars. To achieve optimal yield potential, the flowering dates of the cultivars within a given orchard must overlap significantly and be coincident with a period of weather that is favorable for bee flight and pollination activity. Following pollination, the almond fruit grows rapidly from March until June, undergoes conversion of nut sugars to proteins and oils, and typically reaches fruit maturity in late July–August, depending upon the local environment. Fruit harvest typically occurs from late July through September^2^.

Two methods have been widely used for crop yield prediction: process-based “crop models” and statistical “machine learning models.” The “crop model” approach forecasts yield by simulating crop growth, nutrient cycling as well as water and energy balance on regular time steps (e.g., daily), driven by environmental factors. Crop Model estimations are carried out by using the physiological characteristics of plants. Widely used crop models include CERES-Maize model (Hodges et al., 1987), CROPGRO-soybean model (Jagtap and Jones, 2002), SALUS model (Dzotsi et al., 2013), APSIM model (Keating et al., 2003), and SWAT (Srinivasan et al., 2010). Although the simulations are based on the known principles and processes that determine crop productivity, these mechanistic models require extensive input data such as cultivar, management, and soil conditions (Cai, 2017). More importantly, the model calibration is often rather challenging due to the complexity of the processes, limited availability of field data across a wide range of environmental gradients, and a large number of uncertain input parameters (Lobell and Burke, 2010). Machine learning-based models, in contrast, aim to build empirical predictive algorithms using historical data from multiple sources. Predictions derived from this approach are not directly based on known physiological mechanisms that determine plant growth, and thus have the advantage of forecasting the yield without relying on the specific parameters for individual crops (Medar and Rajpurohit, 2014; Droesch, 2018). Some studies have also compared and combined the process-based models with statistical machine learning approaches to analyze the climate impacts on crop productivity (Lobell and Asseng, 2017; Roberts et al., 2017).

Relatively simple statistical models have been developed to predict year-to-year variability of yield for several California crops, in response to climate change, at either the county- or state-level (Lobell and Field, 2007; Lobell and Cahill, 2011). The development of these models relies on annual CASS yield statistics (California Agricultural Statistics Service [CASS], 2018). For example, Lobell and Field (2007) investigated the climate and statewide almond yield relationship during 1980–2003. These studies provide insight into the potential impact of future climate change on perennial cropping systems in California. However, two limitations still remain: (1) the spatial size of prediction unit is quite large, at either the county or state level, while the prediction at finer spatial scale (orchard/field level), which is critical for growers to properly manage their farm resources (e.g., water and nitrogen), hasn’t been explored; (2) the models developed were built purely based on climate variables, while other yield determinants, such as planting years, canopy characteristics, and tree vigor, were not considered.

Canopy size and crop vigor are key determinants of crop yield. Satellite remote sensing makes it possible to characterize the canopy structure and crop vigor at medium to high spatial resolutions at different time scales, and thus has great potential for crop yield analysis (Ferencz et al., 2004; Rembold et al., 2013; Johnson, 2014; Sibley et al., 2014; Guan et al., 2017). Multiple vegetation indices (VIs) from MODIS data, for example, were used to predict the yield for maize and soybean at the county level in the Midwestern United States (Bolton and Friedl, 2013). Time series NDVI extracted from MODIS data were applied to estimate yield for corn and soybean in the “corn belt” in the United States (Li et al., 2007).

Various statistical approaches including linear regression models (Lobell and Burke, 2010; Bolton and Friedl, 2013; Ramesh and Vardhan, 2015), and machine learning models such as artificial neural network (Kaul et al., 2005; Dahikar and Rode, 2014) and support vector regression (Jaikla et al., 2008; Brdar et al., 2011), have been developed for crop yield prediction. However, most of these approaches rely on one regression model for the prediction, and are therefore subject to overfitting when the training data is limited (Pal, 2007). In the machine learning community, there is an increasing interest in combining several base learning models into one predictive model in order to improve the model generalization ability (Chi et al., 2009; Zhou, 2009; Zhang and Crawford, 2015). By combining multiple learners, the errors of a single model will likely be compensated by others, and thus help improve the robustness and accuracy of the prediction. Moreover, in some cases, the optimal hypothesis may be outside the space of any single model. The search space may be extended by combining different models, and thus a better fit to the data space can be achieved (Pal, 2007; Sagi and Rokach, 2015). Bagging and boosting are among commonly used techniques in this context (Skurichina and Duin, 2002). Bagging uses bootstrap sampling to obtain the data subsets for training and generating the base learners in parallel. Recently, a representative bagging model – Random forest (RF) has been investigated for crop yield prediction (Everingham et al., 2016; Jeong et al., 2016), and better performance was found when comparing to the traditional multiple linear regression approach (Jeong et al., 2016). Boosting generates the base models in a sequential way in order to exploit the dependence between the base learners. Stochastic gradient boosting (SGB) is a representative boosting method which also uses the decision tree as the base learner. In contrast to RF which builds many independent trees, SGB generates the subsequent tree by learning from the mistakes of the previous one, and thus usually performs better than RF (Lawrence et al., 2004). To date, the SGB algorithm approach has only been applied in very few applications in agronomy (Freeman et al., 2015; Forkuor et al., 2017), and to the best of our knowledge, it has not been applied for crop yield prediction.

Our goal was to build data-driven machine learning models for orchard-level California almond yield prediction at both early and mid-season. The availability of a large quantity of yield records for individual orchards in the Central Valley of California allowed us to develop accurate models by incorporating all the relevant variables that are expected to affect the yield. For this purpose, informative variables were first extracted from different data sources, including the consultation with experienced researchers and growers, orchard statistics from grower provided data, climate variables and remote sensing metrics derived from public and private data-sources. Machine learning models were then built to predict the final end-of-year yield in March (early season) and June (mid-season). To evaluate the model performance, the prediction results for the years 2010–2017 were compared with the yield records provided by the growers. Finally, the most predictive variables were selected from the models and used to analyze the spatial and temporal yield variation patterns.

## Materials and Methods

### Study Area

**FIGURE 1:**
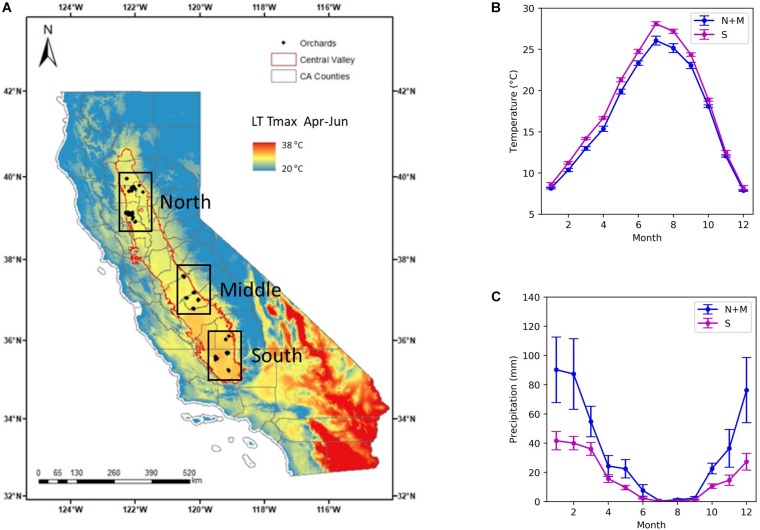
Almond orchard study area in California’s Central Valley. **(A)** Historical yield records from growers were collected for a total of 185 almond orchards (diamond), located in three subregions: north, middle, and south (black boxes). The long-term mean maximum temperature (LT Tmax) during April–June from 1900 to 2009 is shown in the background. Also shown are **(B)** Mean monthly temperature and **(C)** monthly precipitation averaged over northern and central (blue) and southern subregions (magenta).

We focused on the almond orchards located in the Central Valley of California, where we have collected the historical yield and other ancillary data from eight growers managing a range of orchards of different ages (Figure 1A). The Central Valley of California is a vast agricultural region drained by the Sacramento and San Joaquin Rivers. It is California’s most productive agricultural region and one of the most productive agricultural regions in the world, providing more than half of the fruits, vegetables and nuts grown in the United States (House Committee on Natural Resources, 2015). Most of the valley lies close to sea level with a very low relief. Climate is characterized by hot and dry summer and mild and wet winter. The long term mean temperature from June to August is 25°C for the north and middle subregions, averaged over 20 years from 1990 to 2009, and 27°C for the south, while November–December–January mean temperature is about 9.5°C for all the regions (Figure 1B). The majority of rainfall occurs in winter and spring from November to April (Figure 1C). The northern and middle subregions receive greater precipitation, with a mean annual precipitation (MAP) of 426 mm, than the southern region, with MAP of 200 mm (Figure 1C). In order to build models to predict the almond yield for individual orchards, we collected data from three sources, including (1) orchard statistics from grower data; (2) climate and weather variables from station and gridded weather data; and (3) canopy and vegetation indices from remote sensing imagery. The detailed descriptions for each data source and the extracted variables are, respectively, discussed in the following Sections “Grower Data, Weather Data, and Remotely Sensed Data,” and a complete summary is shown in Table 1.

**TABLE 1:** A detailed summary of the input variables for early-/mid-season SGB models.

**Data source**	**Raw data**	**Model input variables**	**Variable name**	**Pearson correlation**
**Grower data**				
Yield records from 8 major almond growers in the Central Valley of California.	Planting year Historical yield Cultivar variety and composition	Age Previous two years’ yield Cultivar percentage	Age Pre 2Y Yld, Pre 1Y Yld CulP 1, …., CulP 20	−0.16^*^ 0.53^*^, 0.53^*^ −0.27∼0.25
**Weather data**				
CA-BCM data with 270 m spatial resolution for years 1990–2016^1^;	Monthly mean daily maximum temperature (Tmax); Monthly mean daily minimum temperature (Tmin); Monthly accumulative precipitation (PPT)	Current year monthly Tmax and Tmin from January to June^2^, and PPT from January to March	Tmin, Tmax, PPT January Tmin, Tmax, PPT February Tmin, Tmax, PPT March *Tmin, Tmax April* *Tmin, Tmax May* *Tmin, Tmax June*	−0.06^*^, −0.06, −0.22^*^ −0.17^*^, 0.17^*^, −0.27^*^ 0.05^*^, 0.21^*^, −0.30^*^ 0.25^*^, 0.30^*^ 0.17^*^, 0.12^*^ 0.17^*^, 0.21^*^
		Previous year summer mean temperature averaged over July and August	Pre Tmean July–August	0.36^*^
		Long-term mean seasonal Tmax, Tmin, PPT (averaged over 1990–2009 for each season^3^).	LT Tmin, Tmax, PPT January–March LT Tmin, Tmax, PPT April–June LT Tmin, Tmax, PPT July–September LT Tmin, Tmax, PPT October–December	0.26^*^, 0.50^*^, −0.58^*^ 0.43^*^, 0.60^*^, −0.61^*^ 0.38^*^, 0.49^*^, −0.57^*^ −0.31^*^, 0.51^*^, −0.58^*^
CIMIS station data for years 2009–2017	Hourly temperature	Winter chilling portions calculated by the Dynamic Model	ChillP	−0.14^*^
**Remote sensing imagery**				
NAIP aerial imagery from 2016 with 0.6m resolution	NAIP RGB imagery acquired in 2016	2016 canopy cover percentage	CCP	0.20^*^
Landsat satellite imagery with 30 m resolution years 2009–2017	Landsat multispectral imagery every 16 days	Previous year annual maximum NDVI and EVI; *Current year June average EVI*	Pre Max NDVI Pre Max EVI *June Mean EVI*	0.13^*^ 0.31^*^ 0.35^*^

^1^2017 Tmax, Tmin and PPT were collected from the original PRISM data archive. ^2^Variables used only for mid-season prediction models were shown in Italic. ^3^The four seasons are separated as (1) January–March, (2) April–June, (3) July–September, (4) October–December. ^*^Represents for *p* < 0.05.

### Grower Data

A critical component for building a yield prediction model at the individual orchard block level is the availability of a database of historical yield records drawn from a wide diversity of almond orchards. We collected those data up to 2017 from eight major almond growers, representing 8143 hectares of almond production areas. A total of 185 orchards are distributed across the major production regions in the Central Valley, with 68 orchards in the northern region, 43 and 74 orchards in the central and southern region (Figure 1). The time span of recorded yield data varied by orchard, and the earliest record dated back to 2005. Overall, about 58% of these orchards had more than 8 years of yield data.

**FIGURE 2:**
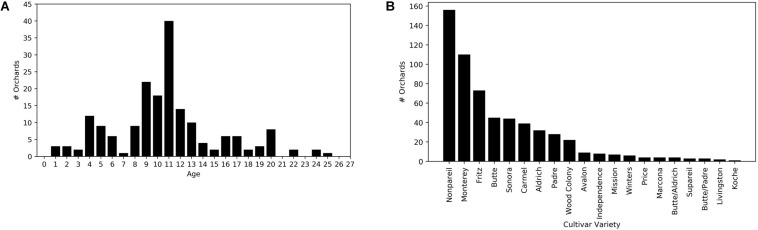
Distributions of **(A)** orchard age in 2017 and **(B)** cultivars for the study orchards (*N* = 185).

Growers also provided several other orchard-specific attributes, such as the field boundary, the orchard size, the year when an orchard was planted, planting density, cultivar varieties and areas for each variety, and other ancillary information related to management practices. The majority of orchards were planted around 2006–2008. By 2017, the median age of all study orchards was 11, and 80 % of orchards were mature, between 7 and 17 years old (Figure 2A).

We also extracted the cultivar percentages, and the associated yields for each cultivar. We further aggregated the reported cultivar-level yields to the total yield for each orchard, which served as the independent variable. A combination of cultivars, rather than single cultivar, are typically planted within each orchard for cross-pollination purposes (Asai et al., 1996). Cultivars may have different bloom timing and bloom overlap which is a critical factor for securing pollination (Asai et al., 1996). Non-pareil and Carmel are two commonly planted cultivars utilized because they have good bloom overlap and therefore are excellent pollinizers for each other (Asai et al., 1996). A total of 20 different cultivars were included across all sample orchards with Non-pareil, Monterey and Fritz the dominant cultivars (Figure 2B). For model development, we constructed an array of 20 elements to represent the cultivar percentage for each one of the 20 cultivars across all orchards. Thus, an orchard with 50% Non-pareil and 50% Monterrey, has a cultivar array of 0.5 for the Non-pareil and Monterrey elements but zeros for the remaining elements associated with the other 18 cultivars.

### Weather Data

We extracted climate and weather variables that were expected to influence the yield for each orchard. The primary meteorological variables from 1990 to 2017 were based on the monthly gridded Parameter elevation regressions on independent slopes model (PRISM) dataset which has 800 m spatial resolution (Daly et al., 2008). This dataset was spatially downscaled to 270 m using the California basin characterization model (CA-BCM) (Flint and Flint, 2014), a regional water-balance model to simulate hydrologic responses to climate. The monthly mean daily maximum temperature (Tmax), minimum temperature (Tmin) and monthly total precipitation (PPT) were collected from the CA-BCM data archive. Three categories were used, including: (1) monthly Tmin, Tmax from January to June, and monthly PPT from January to March in the target year, (2) previous year summer temperature, calculated by averaging the Tmax and Tmin from July and August, (3) long-term mean seasonal meteorological variables averaged over years 1990–2009 for each season (JFM, AMJ, JAS, OND) (Table 1).

Winter chill is also one of the defining characteristics for tree crop production, and it can be quantified with two different mathematical models, including the Chilling Hours Model (Chandler, 1942), and the Dynamic Model (Erez and Fishman, 1998). The Chilling Hour Model has been found to be very sensitive to temperature increases, and its accuracy is likely to decrease, especially in warm growing regions (Luedeling, 2012). On the other hand, the Dynamic Model, a more complex but also more accurate approach, can better characterize the real chilling conditions (Luedeling, 2012). We here therefore calculated the preceding winter chilling portions following the Dynamic Model. The hourly temperature data were obtained from the California Irrigation Management information system (CIMIS) network (California Irrigation Management Information System [CIMIS], 2018). CIMIS currently operates a network of 164 automated stations across California, collecting weather data on a minute-by-minute basis and the data are archived as hourly averages for each location. For each orchard, the weather data from its closest CIMIS station were used for deriving the winter chill variable for each year and for each orchard. A total of thirteen CIMIS stations were used in this study.

### Remotely Sensed Data

We derived the canopy cover percentage (CCP) and vegetation indices (VIs) from remote sensing imageries for each individual orchard. The 2016 USDA National Agriculture Imagery Program (NAIP) imagery with a spatial resolution of 0.6 m, typically acquired during the crop growing season, was used in this study. We first classified this aerial imagery of 4 spectral bands (red, green, blue, and near infrared) into three classes (canopy cover, bare soil, and shadow), using support vector machine (SVM), a supervised classification approach for each orchard. The CCP was then estimated as a ratio of canopy area over the total orchard area based on the high-resolution classification map.

We derived the mean VIs including the widely used normalized difference vegetation index (NDVI) and enhanced vegetation index (EVI) from time series of Landsat satellite observations. The Landsat level-2 surface reflectance product (Landsat-5: 2009–2011, Landsat-7: 2012, and Landsat-8: 2013–2017) archived in Google Earth Engine was used in this study, as it has been processed by the Landsat Ecosystem Disturbance Adaptive Processing System (LEDAPS). We first conducted a pixel-based quality check to screen and filter out the poor-quality surface reflectance values using cloud mask and quality assessment (QA) information in the Landsat metadata. This step eliminated the observations contaminated by clouds from the whole Landsat archive, and also the default fill values in the strip gaps due to the Landsat-7 ETM+ scan line corrector (SLC) failure since May 31, 2003 (Chen et al., 2017). NDVI and EVI values were then calculated from the retained reflectance in the multispectral bands for each pixel. Finally, we derived both annual maximum and monthly average values for each VI.

### Yield Prediction Models and Accuracy

We tested different machine learning approaches including linear regression, support vector regression, neural network, RF, and finally chose SGB (Freeman et al., 2015) for both early- and mid-season yield prediction, due to its strong predictive power (Friedman, 2001). SGB is a machine learning technique for regression and classification problems (Lawrence et al., 2004; Forkuor et al., 2017). In SGB, simple decision trees are often used as the base learners, and each successive tree is fitted to minimize the prediction residuals computed based on all preceding trees. At each iteration, a subsample is drawn at random from the full training dataset, to reduce the correlation between the trees in sequence in the model. The randomly selected subsample is then used, to fit the base learner. In this study, we randomly select 50% of the training data to build each individual tree which has a maximum depth of ten. The ensemble of trees continues to grow as the iteration proceeds, until a fixed total number of trees (set at 100 for this study) are added to the ensemble of the trees in the model. The final prediction is achieved by adding together the predictions of each decision tree, and the contribution of each tree to this sum is weighted by a fixed learning rate (set at 0.1 for this study).

We used the “scikit-learn” package from Python (Pedregosa et al., 2011) to build various SGB regression models with different levels of complexity, depending on the availability of the input data. The dependent variable is the almond yield for a particular year during 2010–2017 of an individual orchard. We explored a suite of predictors including orchard statistics from growers (i.e., historical yields, orchard age, and cultivar percentage), weather and climate variables, and canopy attributes from remote sensing imagery, as shown in Table 1. Multi-year data were pooled to train models because this increased the quantity and diversity of the training data. Since the historical yields were found to be a good indicator of almond yield, we first explored their utility in the prediction and found that including more than 2 years of yields in the preceding years did not increase the prediction accuracy for the target year (Supplementary Table S1). We therefore used only the previous 2 years of yields as one type of predictors, e.g., 2017 yield was predicted by using individual year 2015 and 2016 yields as input variables. For a particular predicting year, a small number of orchards do not have the previous two years’ yield records, and therefore were removed from the model building and validation process. The remaining samples were pooled, resulting in 990 sample points (orchard and year). For each orchard sample, we paired the target year’s yield with the corresponding input variables for that particular year.

Correlations were computed between (1) each input variable with the yield (Table 1), and (2) any two input variables (Supplementary Figure S1). The results show that some input variables have strong positive correlations with the almond yield such as the long-term seasonal maximum temperatures, while some exhibit strong negative correlations with the yield such as the long-term seasonal precipitation. Some input variables are also highly correlated with each other, such as the long term seasonal Tmin, Tmax and PPT and the remote sensing metrics (Supplementary Figure S1).

**FIGURE 3:**
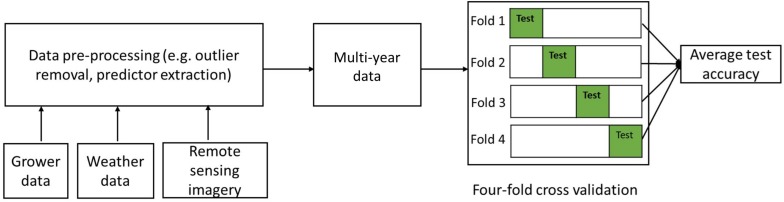
Flowchart of the four-fold cross validation modeling framework.

To further test the robustness of the developed models, we adopted a four-fold cross-validation strategy for model building and testing (Zhang et al., 2017; Lyons et al., 2018), as shown in Figure 3. Specifically, the 990 samples were randomly partitioned into four subsets of equal size, resulting 247 samples in three subsets and 249 in one subset. In each round, one of the four subsets was retained as the independent test set, whereas the remaining three subsets were used as training data. We evaluated the model performance, by quantifying the following metrics based on the testing data for each round, including (1) the root mean squared error (RMSE), (2) the coefficient of determination (*R*^2^), and also (3) the ratio of performance to interquartile distance (RPIQ), which is defined as interquartile range of the observed values divided by the RMSE (Maurel et al., 2010). The RPIQ takes account of both the prediction error and variation of observed values, and therefore it is more objective than the RMSE and more easily to compare among models. The greater the RPIQ, the stronger predictive capacity of the model (Maurel et al., 2010). The final accuracy was summarized by averaging these metrics from the four rounds and the corresponding standard deviations were also recorded.

We recognized that the historical yields and cultivar information sometimes are hard to collect for all individual orchards, which can limit the prediction over a large region. In addition to full models, we therefore also developed two additional reduced models: (1) excluding last 2 years’ historical yields, and (2) excluding both historical yields and cultivar percentage from the input data. We then evaluated the model performance in a similar way as described above for the full models. These models will allow for the yield prediction when subsets of information are not available.

### Determinants of Almond Yield

We evaluated how important the predictor variables were in predicting orchard-level yield. In the “scikit-learn” package from Python, the variable importance was implemented based on the metric “mean decrease impurity” (or called “mean decrease Gini”) (Breiman et al., 1984), with higher values indicating that a variable is more important in producing accurate predictions (Breiman et al., 1984). We also used partial dependence plots (PDPs) (Molnar, 2018) to further understand how each of the top important variables affected the yield. A PDP is a graphical representation, which aims to show the influence of the variable of interest on the predicted outcome (yield). Partial dependences work by marginalizing the machine learning model over the distribution of all other variables so that the remaining function shows the relationship between the targeting variable and the yield (Molnar, 2018). We investigated the main factors that drove the spatial and temporal variations in almond yield. Ensemble trees within a SGB model are much more difficult to interpret due to the complexity of the multiple trees, although they have been reported to outperform the single tree in terms of the prediction accuracy (Wang et al., 2015). To further examine how the spatial and temporal yield variations are driven, we also built a single decision tree model, based on the important variables and mature orchards, as the splitting nodes in the single tree model can help us better understand the roles of the main factors in terms of affecting the yield variation. Single tree structure can also provide important insights about the spatial-temporal yield patterns.

## Results

### Almond Yield Variability Based on the Growers’ Data

**FIGURE 4:**
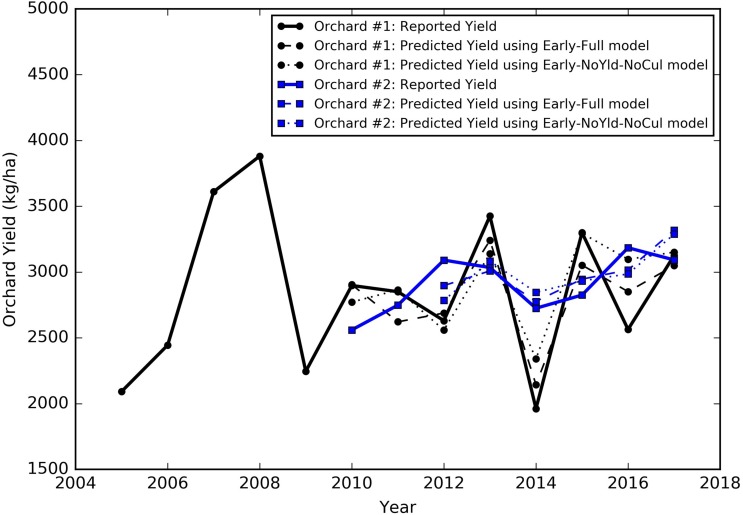
Historical yield patterns for two sample orchards, Orchard #1 is located in Colusa County, planted in 2001 (black), and Orchard #2 is located in Kern County, planted in 2006 (blue). The corresponding early season predictions were also shown as (a) dashed lines by the full model and (b) dotted lines by the reduced model excluding the historical yield and cultivar composition from predictors.

The yield of almond orchards typically has a large inter-annual variation, even after reaching maturity as shown by grower reported yield records. For illustrative purposes, we chose two mature orchards located in different regions, that had at least 8 years of yield records, one in the north region (Colusa County) and the other in the south region (Kern County), to show typical yield dynamics. The yield of the northern orchard, planted in 2001, increased steadily at young ages, e.g., from 2092 kg/ha in 2005 to 3881 kg/ha 2008 (Figure 4). It then started to fluctuate from year to year, with the lowest yield of 1961 kg/ha in 2014 and the highest of 3426 kg/ha in 2013. For the southern orchard, planted in 2005, similar patterns of increases in the yield were found when the orchard is younger than 7 years old (from 2010 to 2012). Its yield reached 3090 kg/ha in 2012 (Figure 4), and then, it declined to 2723 kg/ha in 2014, and increased again to 3184 kg/ha in 2016. However, in general, the yield variation for this orchard (Kern County) is smaller than the orchard in Colusa County.

**FIGURE 5:**
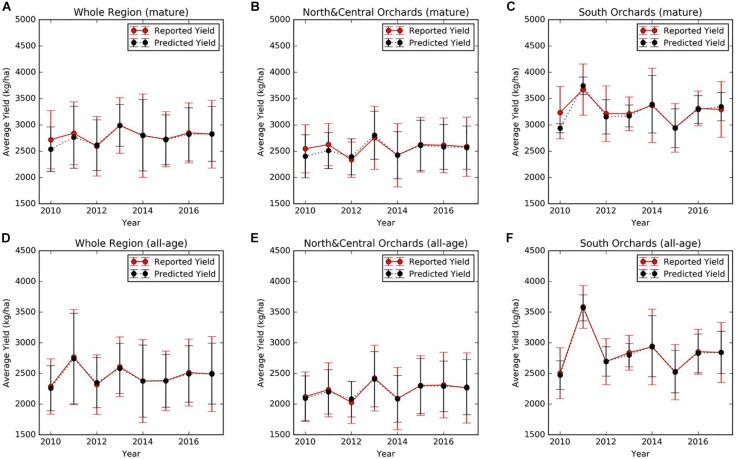
Mean annual reported almond yield and early-season predictions averaged **(A)** over mature (7–17 years old) orchards and **(D)** over all orchards within the study area. Similar results were presented here for **(B,E)** orchards in the northern and central region and **(C,F)** for southern orchards, respectively. The vertical lines represented the standard deviation among orchards.

At the regional scale, we summarized over all mature orchards (7–17 years old). The mean almond yield also showed large variation from year to year, ranging from 2718 ± 554 kg/ha in 2010 to 2988 ± 530 kg/ha in 2013 (Figure 5A). It is also evident that the yield varies spatially at the regional scale. For example, the lowest and highest yield were 2339 ± 335 kg/ha in 2012 and 2755 ± 599 kg/ha in 2013, averaged over all mature orchards in the north and central regions (Figure 5B). In the southern part of Central valley, yield of orchards of similar ages is much higher than the north and central regions, ranging from a low of 2945 ± 463 kg/ha in 2015, to a high of 3674 ± 487 kg/ha in 2011, respectively (Figure 5C).

### Model Performance

We first compared the performance of five most widely used machine learning approaches (Table 2), using all input variables (full models). For both the early- and mid-season predictions, the results showed that the RF and the SGB outperformed the other three models. For example, they achieved an *R*^2^ around 0.7, while the other three models obtained an *R*^2^ of less than 0.6. The SGB models were found to have better performance as shown by their higher *R*^2^ and RPIQ values and smaller RMSE, than the RF models (Table 2). Therefore, we focused on the SGB approach for the following analysis. Also, the strengths and weakness of SGB are further compared with other approaches in the section “Discussion.”

**TABLE 2:** Comparison of the performance of five machine learning approaches for orchard-level almond yield prediction, when using the full set of input variables.

**Prediction**	**Machine learning**		**RMSE**	
**time**	**approach**	***R*^2^**	**(kg/ha)**	**RPIQ**
Early season	Linear regression	0.58 (0.04)	422 (4.1)	2.19 (0.19)
	Support vector regression	0.51 (0.04)	460 (16.5)	2.01 (0.16)
	Artificial neural network	0.50 (0.05)	474 (26.1)	1.96 (0.16)
	Random Forest	0.69 (0.04)	364 (14.8)	2.55 (0.28)
	**Stochastic gradient boosting**	**0.71 (0.04)**	**352 (15.2)**	**2.64 (0.33)**
Mid-season	Linear regression	0.59 (0.05)	416 (6.1)	2.23 (0.22)
	Support vector regression	0.52 (0.04)	453 (15.5)	2.05 (0.17)
	Artificial neural network	0.48 (0.04)	473 (7.6)	1.96 (0.15)
	Random Forest	0.69 (0.04)	365 (13.9)	2.54 (0.27)
	**Stochastic gradient boosting**	**0.71 (0.04)**	**355 (12.3)**	**2.62 (0.29)**

*A four-fold cross-validation strategy was used for model building and testing; both mean values and standard deviations (in parenthesis) of *R*^2^, RMSE, and RPIQ are presented here based on the comparison of the prediction and the independent testing data.*

For the two individual orchards mentioned in Section “Almond yield variability based on the growers’ data,” for example, the yield predicted by the SGB early season model captured the variation from year to year, similar to the historic yield record reported by the growers (Figure 4, Early Full model). Specifically, the median departure from the reported orchard yield across multiple years was 185 kg/ha for the northern orchard in Colusa County and 145 kg/ha for the southern orchard in Kern County. The smallest difference between the prediction and the ground truth was around 7 kg/ha for the northern orchard and 26 kg/ha for the southern orchard.

**FIGURE 6:**
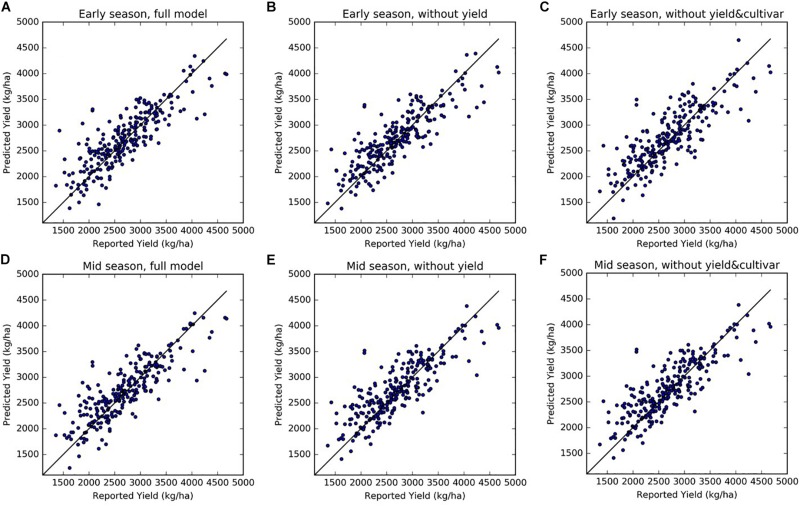
Scatter plots of predicted vs. reported yields, from three sets of models for early season (top panels) and mid-season predictions (bottom panel): **(A,D)** full models, **(B,E)** reduced model excluding historical yields as predictors, and **(C,F)** reduced models excluding both historical yield and cultivar composition information. **(A)**: Early Full; **(B)** Early NoYld; **(C)** Early NoYld-NoCul; **(D)**: Mid-Full; **(E)** Mid-NoYld; **(F)** Mid-NoYld-NoCul. The predicted values shown here were from the reserved testing dataset in one round of the cross-validation (*N* = 247).

The four-fold cross validation with independent testing data showed that the full SGB models for both early and mid-season predictions performed well and achieved similar accuracies (Table 3). In each round of the four-fold cross validation, one of the four subsets was retained as the test set, whereas the remaining three subsets were used as training data. Therefore, we evaluated the model performance on the test set at each round. The final accuracies were reported by averaging the results from each round, and therefore there is a standard deviation. When using all 49 input variables, the early season full model explained 71% of the variance in the reserved testing data and the corresponding RPIQ exceeded 2.6 on average, over the four rounds of model building and testing. The model results were robust, as shown by the small standard deviations of *R*^2^, RPIQ, and RMSE across four rounds of cross validation. The mid-season SGB full model had similar performance, with an *R*^2^ of 0.71 (± 0.04) and an RPIQ of 2.62 (± 0.29). As an example result from one round run of the four-fold cross validation, Figures 6A,D further showed the good agreement between prediction and growers-reported yield in the reserved testing dataset (*N* = 247) for both the early and mid-season using the full models. The average time series early season predictions using the full model are shown for mature orchards only (Figures 5A,B,C) and also for all-age orchards (Figures 5D,E,F), across different regions. In general, the predicted yields aligned well with the reported yields.

**TABLE 3:** Performance of the Stochastic Gradient Boosting (SGB) approach in predicting the almond yield at individual orchards, using different set of input variables, as shown by the statistics from a four-fold cross-validation.

				**# Input**		**RMSE**	
	**Prediction time**	**Input variables**	**Model name**	**variables**	***R*^2^**	**(kg/ha)**	**RPIQ**
**1**	**Early season**	**All variables**	**Early Full**	**49**	**0.71 (0.04)**	**352 (15.2)**	**2.64 (0.33)**
2	Early season	Without historical yields	Early NoYld	47	0.70 (0.05)	355 (17.6)	2.62 (0.32)
3	Early season	Without historical yields and cultivar percentage	Early NoYld-NoCul	27	0.68 (0.04)	370 (6.2)	2.51 (0.26)
4	Early season	Only important variables	Early Imp	8	0.67 (0.04)	375 (18.2)	2.48 (0.26)
**5**	**Mid-season**	**All variables**	**Mid-Full**	**56**	**0.71 (0.04)**	**355 (12.3)**	**2.62 (0.29)**
6	Mid-season	Without historical yields	Mid-NoYld	54	0.70 (0.05)	360 (17.2)	2.59 (0.34)
7	Mid-season	Without historical yields and cultivar percentage	Mid-NoYld-NoCul	34	0.69 (0.05)	364 (15.9)	2.56 (0.33)
8	Mid-season	Only important variables	Mid-Imp	10	0.68 (0.04)	371 (6.3)	2.50 (0.25)

We also tested two sets of reduced models by using subsets of the input variables: (1) without using last 2 years’ historical yields as input (NoYld); (2) without using historical yields and without cultivar percentage (NoYld-NoCul). We found that removing the historical yields and cultivar percentage from the input variables did not significantly reduce the accuracy for both early and mid-season predictions (Table 3). For the early season prediction, the model without historical yields had an *R*^2^ of 0.70 (± 0.05), and an RPIQ of 2.62 (± 0.32); after further removing the cultivar information as one of the predictors (Early NoYld-NoCul), the *R*^2^ slightly decreased to 0.68 (± 0.04), and the RPIQ decreased to 2.51 (± 0.26). For the previously mentioned two sample orchards (Figure 4), the predicted yield by the Early NoYld-NoCul model were only slightly worse than the predictions using the full model. Similar to the full models, the scatter plots (Figures 6B,C,E,F) for the reduced models also showed that the predicted yield agreed well with the yields reported by growers. This result demonstrated that the reduced models can be directly applied to predict yield for orchards reasonably well, when the cultivar and historical yield information is lacking.

### Variable Importance

**FIGURE 7:**
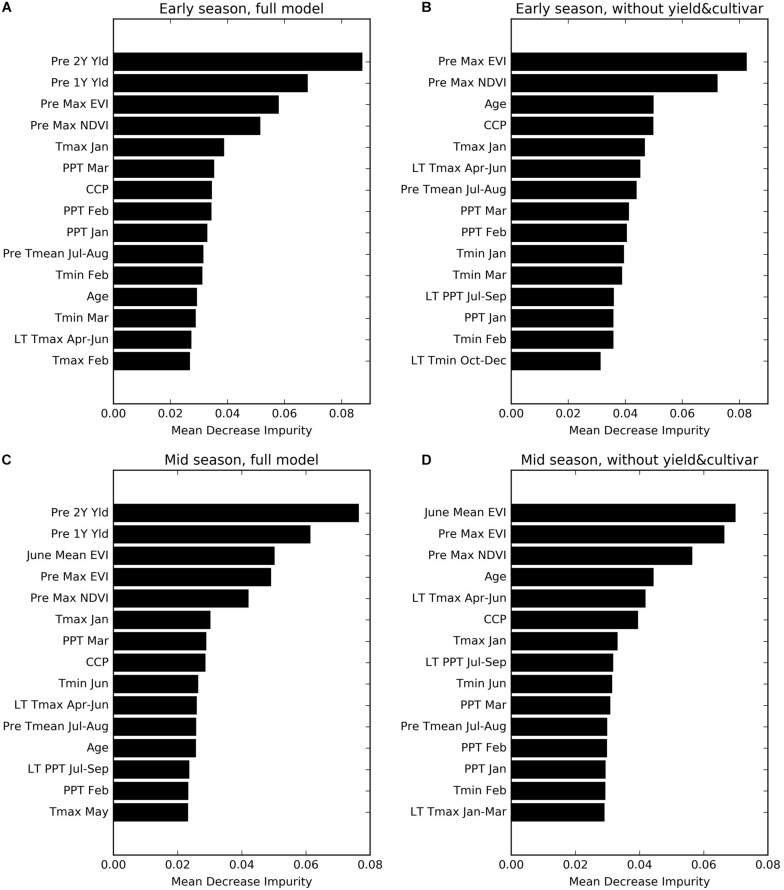
Variable importance for four sets of prediction models **(A)** Early Full; **(B)** Early NoYld-NoCul; **(C)** Mid-Full; **(D)** Mid-NoYld-NoCul. See Table 1 for the detailed variable names.

We found that the previous 2 years’ yields (Pre 2Y Yld, Pre 1Y Yld) were most important, in the full models, for predicting the yield, followed by the annual maximum EVI and NDVI the year before (Pre Max EVI and NDVI) for the early season prediction (Figure 7A), and the mean EVI in June during current year (June Mean EVI) for the mid-season adjustment (Figure 7C), probably due to its representation of emerging canopy vigor. In the reduced models without historical yield and cultivar information, these remote sensing metrics and orchard age become the most important variables (Figures 7B,D). CCP was another important metric for canopy characterization.

The key climate variables that ranked relatively high included the maximum temperature in January (Tmax January), precipitation in March (PPT March), long term mean maximum temperature during April–June (LT Tmax April–June), and previous year summer temperature (PreY Tmean July–August). Although the climate variables from April–June were incorporated into the mid-season models, most of them did not show significant importance for the yield prediction, except for minimum temperature in June (Tmin June), which ranked higher than the others.

Based on the above analysis, to further investigate the effectiveness of those important variables, we also built another two further reduced models for early- and mid-season predictions, respectively. Specifically, only eight variables including Age, PreY Max NDVI, PreY Max EVI, CCP, Tmax Jan, PPT Mar, LT Tmax April–June, and PreY Tmean July–August, were used for the early season prediction model; and only two more variables, June Mean EVI and Tmin Jun, were added for the mid-season prediction. Our results showed that these models with only 8 or 10 variables, including remote sensing metrics, age, and climate variables, were able to predict the yields reasonably well (Table 3, Exp. 4 and Exp. 8). The testing accuracy was slightly reduced, but with a comparable *R*^2^ of 0.67 and an RPIQ of 2.48 for the early season prediction, and an *R*^2^ of 0.68 and an RPIQ 2.50 for the mid-season adjustment, which were similar to the results from the intermediate models in Early NoYld-NoCul (Table 3, Exp. 3) and Mid-NoYld-NoCul (Table 3, Exp. 7).

### Determinants of Almond Yield

**FIGURE 8:**
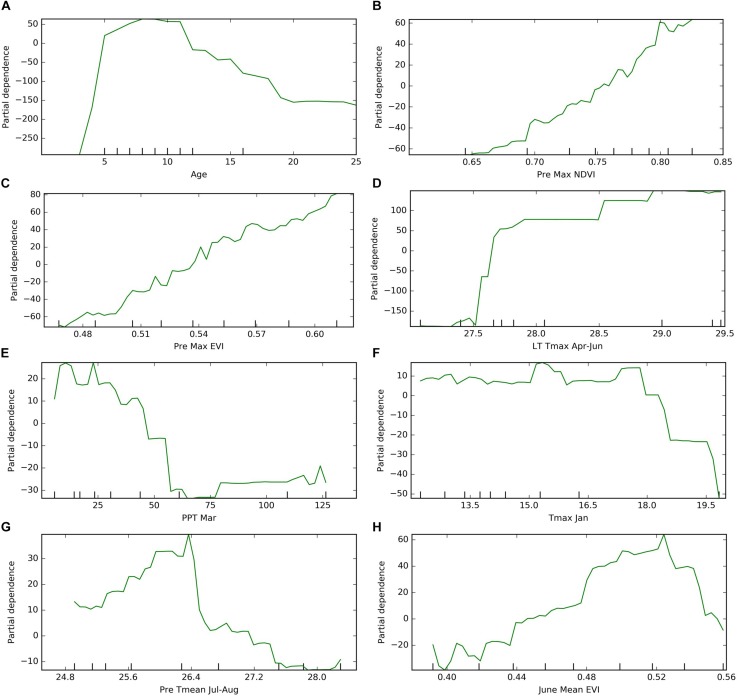
Partial dependence of target year’s yield on **(A)** age, **(B)** previous year maximum NDVI; **(C)** previous year maximum EVI; **(D)** long term maximum temperature in April–June; **(E)** target year March precipitation; **(F)** target year’s January maximum temperature; **(G)** previous year summer temperature; **(H)** target year June mean EVI. ^*^The results were based on “Mid-NoYld-NoCul” model.

The partial dependence analysis was conducted based on the Mid-NoYld-NoCul prediction model. The cultivar composition was not included for this analysis since previous analysis indicated it was not important in yield prediction, and previous yield was also removed as it is highly correlated with age. The resulting PDP plot further confirmed the impact of age on the orchard yield. The plot showed the yield firstly increases dramatically before the tree matures which is around 7 years old, then it drops significantly after 17 years old (Figure 8A). We further removed the age effect by considering only the mature orchards (7–17 years old), to investigate the impacts of other important variables determined in Section “Variable Importance.” We found that yield is positively correlated with the previous year maximum NDVI, EVI, and current year’s June EVI (Figures 8B,C,H).

**FIGURE 9:**
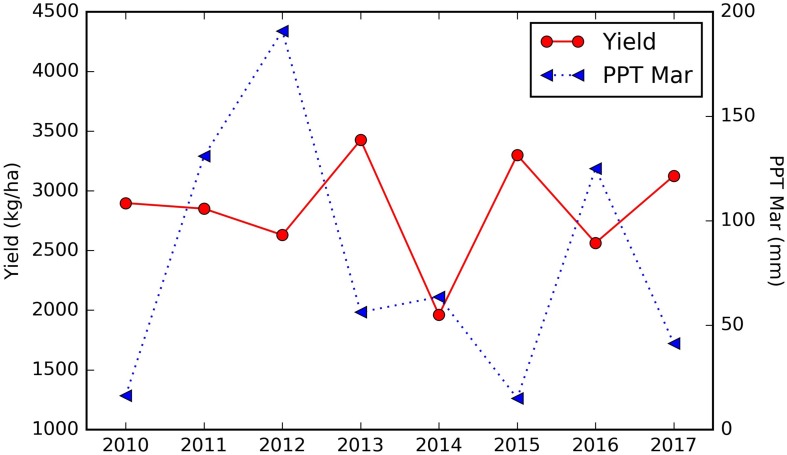
Time series of almond yield reported vs. March precipitation for a sample orchard located in Colusa county, planted in 2001.

Among the important climate variables, it is clear that the long term mean maximum April–June temperature (LT Tmax April–June) enhanced the yield (Figure 8D), especially when it is lower than 28°C. The relatively high yield above the threshold of around 28°C will be further discussed in the Section “Yield Variation Analysis.” Greater precipitation in March (PPT Mar), coincident with the typical almond blooming period, was found to reduce the yield (Figure 8E). For example, for the sample orchard located in Colusa County, the lower yield years (2012, 2014, and 2016) were associated with higher March precipitation, while low precipitation years, e.g., 2013, 2015, and 2017, had relatively higher yield (Figure 9). The maximum temperature in January (Tmax Jan) was negatively correlated to the yield (Figure 8F). In addition, we found that the higher summer temperature during the previous year reduced yield (Figure 8G).

### Yield Variation Analysis

**FIGURE 10:**
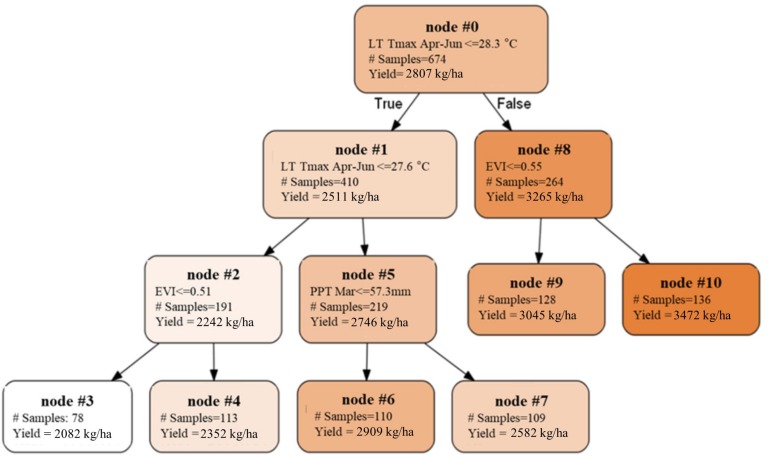
Single decision tree built upon mature orchards using five important variables as predictors. Nodes with higher yields were represented with darker colors.

**FIGURE 11:**
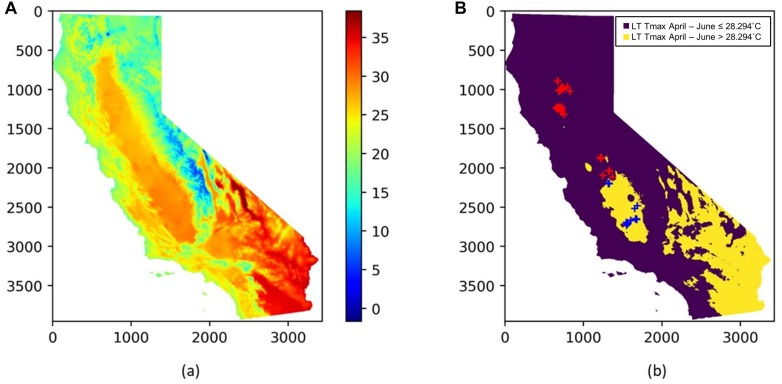
**(A)** Spatial distribution of the long term mean Tmax from April to June (LT Tmax April–June); **(B)** locations of almond orchards in this study overlaid on top of a binary map of LT Tmax April–June with a threshold of 28.294°C; orchards with LT Tmax April–June < = 28.294°C (red cross); orchards with LT Tmax April–June > 28.294°C (blue cross); The binary long term mean April–June temperature map is shown in the background.

To extend the above analysis, and further examine how the spatial and temporal yield variations are driven, we built a single decision tree model, by focusing on the mature orchards (7–17 years old) and using only the most important five climate and remote sensing variables as input variables, including Tmax Jan, PPT Mar, LT Tmax April–June, PreY Max EVI and CCP. The splits and nodes from the resulting single tree structure provided several important insights about the yield patterns, as shown in Figure 10. The first split suggested a threshold of the LT Tmax April–June around 28.3°C, with an average yield of 3265 kg/ha and 2511 kg/ha for areas above or below that threshold, respectively. Interestingly, the spatial distribution of the LT Tmax April–June temperature showed a clear separation of northern and southern orchards (Figure 11), e.g., the LT Tmax April–June temperature is higher than 28.3°C in southern orchards, indicating that long term temperature was the main driver for the spatial variation in the yield.

**FIGURE 12:**
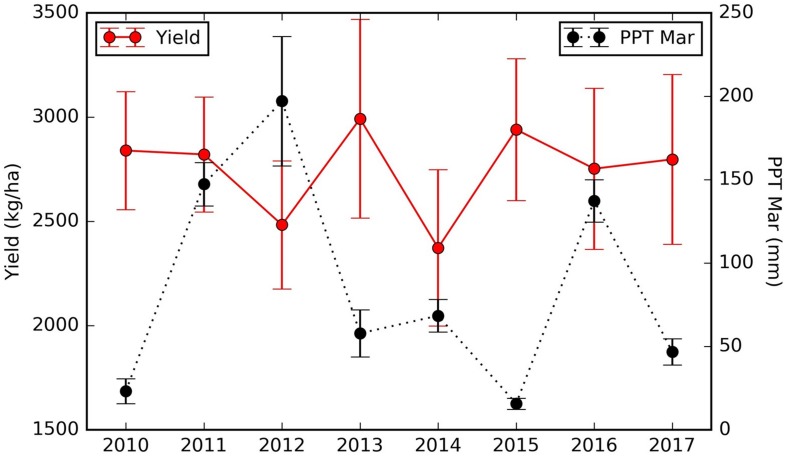
Time series of almond yield and March precipitation for the orchards in node #5 (Figure 10).

We also found that the northern orchards with lower LT Tmax April–June temperature, orchards with canopy cover less than 51% had much lower yield (node #3), 2082 kg/ha on average vs. 2352 kg/ha for denser canopy. Over the areas with intermediate LT Tmax April–June temperature (node #5), orchards and/or years with March precipitation lower than 57.3 mm/mon had a yield of 2909 kg/ha (node #6), about 327 kg/ha higher than those that received larger amount of precipitation in March (node #7). Since these orchards (node #5) are spatially concentrated, the PPT Mar is a strong predictor for the temporal yield variation. Figure 12 shows the time series Yield vs. PPT Mar for the orchards in node #5. For years with lower precipitation (2010, 2013, 2015, and 2017), higher yields were reported. While for the other years with higher precipitation (2011, 2012, 2014, and 2016), lower yields were observed. This contrast pattern indicates that PPT Mar is the main driver for the temporal yield variation, and it has a negative effect for the yield. Also, the tree shows that southern orchards with PreY Max EVI lower than 0.55, on average, had a lower yield (3045 kg/ha, #node 9) than orchards with higher PreY Max EVI (3472 kg/ha, node #10). Also, unlike the climate variables which most affect the temporal yield variation, the remote sensing metrics may reflect more the spatial variability in planting density and management practices.

## Discussion

California’s almond acreage has expanded rapidly in the past 10 years, raising concerns of potentially elevated fertilizer usage and the resulting nitrogen loss to the environment. As the primary determinant of fertilizer N demand is crop yield, improved yield prediction at the orchard block level has become imperative to close the nitrogen management gap.

We here developed machine learning models to predict the almond yield for individual orchards, both at the early season (March) and mid-season (June), using orchard characteristics, climate and weather variables, and the canopy fraction and vegetation indices from remote sensing imagery as primary input data. To our knowledge, this is the first study for the field level yield predictions for the tree crops which typically involve more complex yield determinants than row crops such as soybeans and corn.

### Capabilities of Machine Learning Models

The SGB models were shown to perform well, e.g., the RPIQ ranging from 2.64 for the full model to 2.48 for the reduced model, when predicting the yield early in the season before leaf-out, compared with the independent data. The models were able to explain more than 70% of spatial and temporal variance in the almond yield as observed by growers for individual almond orchards from northern to southern Central Valley. To further evaluate the model performance by treating the “year” as the random variable, we also built the model with data before 2017 (*N* = 824) and tested the model performances on 2017 data (*N* = 166). The early season full model had a slightly poorer performance with an *R*^2^ of 0.62 and an RPIQ of 2.42. The earlier years have fewer training data, the models were less robust and therefore were not included here. As the data set grows the predictive value will undoubtedly increase.

In the current literature, relatively simple statistical models were developed to predict the almond yield only at larger spatial unit, e.g., from the county to the state levels. For the whole state of California, climate variables alone were able to explain 80% of interannual variability in the almond yield from 1980 to 2003 (Lobell and Field, 2007); much larger uncertainties were found at the county-level, with an *R*^2^ of ∼0.25 (Lobell and Cahill, 2011).

The SGB was chosen as our final prediction model based on its stronger prediction performance for this study, when comparing to four other approaches. RF, also had higher prediction accuracy than the other three models. This is probably because combining multiple base learners into one predictive model can help decrease the variance (bagging) and/or bias (boosting) (Sagi and Rokach, 2015). In this study, the SGB method performed better than RF, probably because at each iteration, the boosting strategy focuses directly on the current prediction error and generate a new tree to minimize that error.

### Uncertainties and Future Work

The machine learning based models such as SGB, are capable of finding the complex linkages of yield and environmental variables. However, we recognize that it is possible that the SGB model may also fail, if the individual base learners are not sufficient (Sagi and Rokach, 2015). The historical yield data for individual blocks contributed from a few large almond growers in California, available to us for the first time, made it possible to test the utility of machine learning approach in predicting the yield. However, the accuracy of the data-driven modeling was limited by the relatively small number of samples. We expect that the uncertainties and the robustness of the models will be further improved as we collect more data from the growers in the next couple of years and incorporate other environmental variables such as the bloom information into modeling.

We used the monthly average temperature and precipitation as the predictors, and didn’t consider the potential impacts of short-term (e.g., daily) weather extremes, such as heat waves, on yield. As the weather data at the finer spatial and temporal scales become available, the extreme weather patterns can be quantified and further incorporated into the yield modeling framework for improved accuracy. Moreover, although canopy cover and vegetation index derived from imagery, representing the tree structure and vigor, were included in the models, the bloom condition was not incorporated in our modeling framework due to the scarcity of the data. We have recently developed an automatic approach to directly quantify the bloom condition, and once calibrated, will include it to further reduce the uncertainties in the yield models. Future study may also consider tree-scale measurements which are directly related to final yield, such as flower density or fruit numbers, to further improve the model performance.

In addition, due to the inherent sequential nature, SGB typically needs longer training time than RF, and it is not straightforward to be implemented for parallel computation (Sagi and Rokach, 2015). With the advance of data science, deep learning models, such as convolutional neural network (CNN) has become popular (You et al., 2017), and can be directly applied on the remote sensing imagery, instead of the pre-defined vegetation indices such as NDVI and EVI, to discover the most relevant features which are best for the model building and thus further improve the prediction accuracy.

### Impacts of Canopy, Weather, and Climate on Almond Yield

In this study, we also investigated the influences of the climate variables on yield from both the spatial and temporal domains, and the results have several implications for explaining the yield variation. We found that the previous 2 years’ historical yields are very important variables in controlling the temporal and spatial variation in almond yield. The orchard age becomes more important when the historical yields are not included in the models, especially for younger orchards. Fractional canopy cover and the annual maximum NDVI and EVI, derived from the previous year imagery, are critical for both the early- and mid-season predictions. These remote sensing metrics capture the overall crown cover and green biomass, and thus represents the composite information about planting density, tree and healthy leaves, and management practices. Therefore, the higher vegetation index values, indicating the healthier the tree vigor, the higher yield, as we found from the partial dependence analysis. EVI during June in current year captures the emerging tree vigor, and thus affects the final yield and helps the mid-season prediction.

During the current year, higher maximum temperatures in January were associated with lower yields. On the other hand, large amounts of precipitation in March was associated with a reduction in yields, probably due to its adverse impact on the bee activity and thus pollination which is an important determinant for nut production. The negative impact of March precipitation was more significant for the northern orchards than the southern region, where precipitation is much lower (Figure 1C). In addition, we found that the summer temperature during the previous year negatively affect the almond yield, probably because that the heat stress can reduce net carbon fixation and thus led to lower yield for the next year. Further analysis indicated that southern orchards had higher yield, mostly as a consequence of the warmer weather during April to June. For example, the long-term maximum temperature during April–June was found to be a main driver for the spatial yield variation between the south and north.

This study analyzed the main drivers for the spatial-temporal yield variation based on the developed prediction model recognizing that yield variation may be related to other factors which were not measured and considered in the model, such as the irrigation water use, soil properties, and other management aspects. Further studies are needed to collect and incorporate such information into analysis and explore their effects on the yield.

### Implications of the Yield Prediction Tools

Crop yield prediction is critical for both the scientific community and human society. While publicly available prediction models are available at the state or county level, yield prediction at a finer spatial scale is not available but is imperative for on-farm N management. A main contribution of this work is that we have built machine learning models that are able to predict, with reasonable accuracy, almond yield for individual orchards at two time points of the year. Yield prediction is critical to grower decision making and efficient N management. While the models presented here are promising, it is evident that further refinement is required if the model is to become a primary tool for efficient N fertilization in Almond. Incorporation of larger data sets, additional proximal and remote variables and the development of new analytical methodologies is underway.

We have demonstrated that the reduced SGB models, excluding the historical yields and cultivar percentage as predictors, still produced reasonable yield prediction. Time series remote sensing observations (Chen et al., 2019) have been used to map orchard age, area and location which can be integrated with the model presented here to provide valuable yield prediction even when grower data was not available. The reduced models developed here can therefore be applied at the scale of county or region based solely on orchard mapping and age determination (Chen et al., 2019) and weather and remote sensing data that does not require individual grower input. This is of value to local government and resource management agencies. At the block level, the yield prediction in the early season developed here can be integrated with publicly available nitrogen budget calculator, which has been widely adopted by growers and implemented through the California Almond Sustainability Program website^3^. The availability of a data-driven prediction model with known uncertainties has the potential to provide a scientifically based and independent estimate of yield and hence may improve nitrogen recommendations. The capability to predict yields for almond orchards across the entire state of California also provides quantitative information and guidance to allocate resources, determine almond price targets, and improve market planning.

## Author Contributions

YJ and PB conducted the almond grower data collection. BC processed the remote sensing data. ZZ and YJ performed the data analysis, built the prediction model, and wrote the manuscript. PB and BC edited and reviewed the manuscript. All authors reviewed the manuscript and agreed with the submission.

## Conflict of Interest Statement

The authors declare that the research was conducted in the absence of any commercial or financial relationships that could be construed as a potential conflict of interest.

## Footnotes

1 https://www.waterboards.ca.gov/centralvalley/water_issues/irrigated_lands/

2 https://www.natureseats.com/blog/product-news/item/the-almond-tree-life-cycle-almond-tree-farming

3 https://www.sustainablealmondgrowing.org/
