# Supplementary material for: California Almond Yield Prediction at the Orchard Level With a Machine Learning Approach
Source: Front Plant Sci. 2019 Jul 18;10:809. doi: 10.3389/fpls.2019.00809 (PMC6656960; doi:10.3389/fpls.2019.00809)
Supplement: Supplementary file 1 [file Data_Sheet_1.docx]

Supplementary Material

# Supplementary Figure


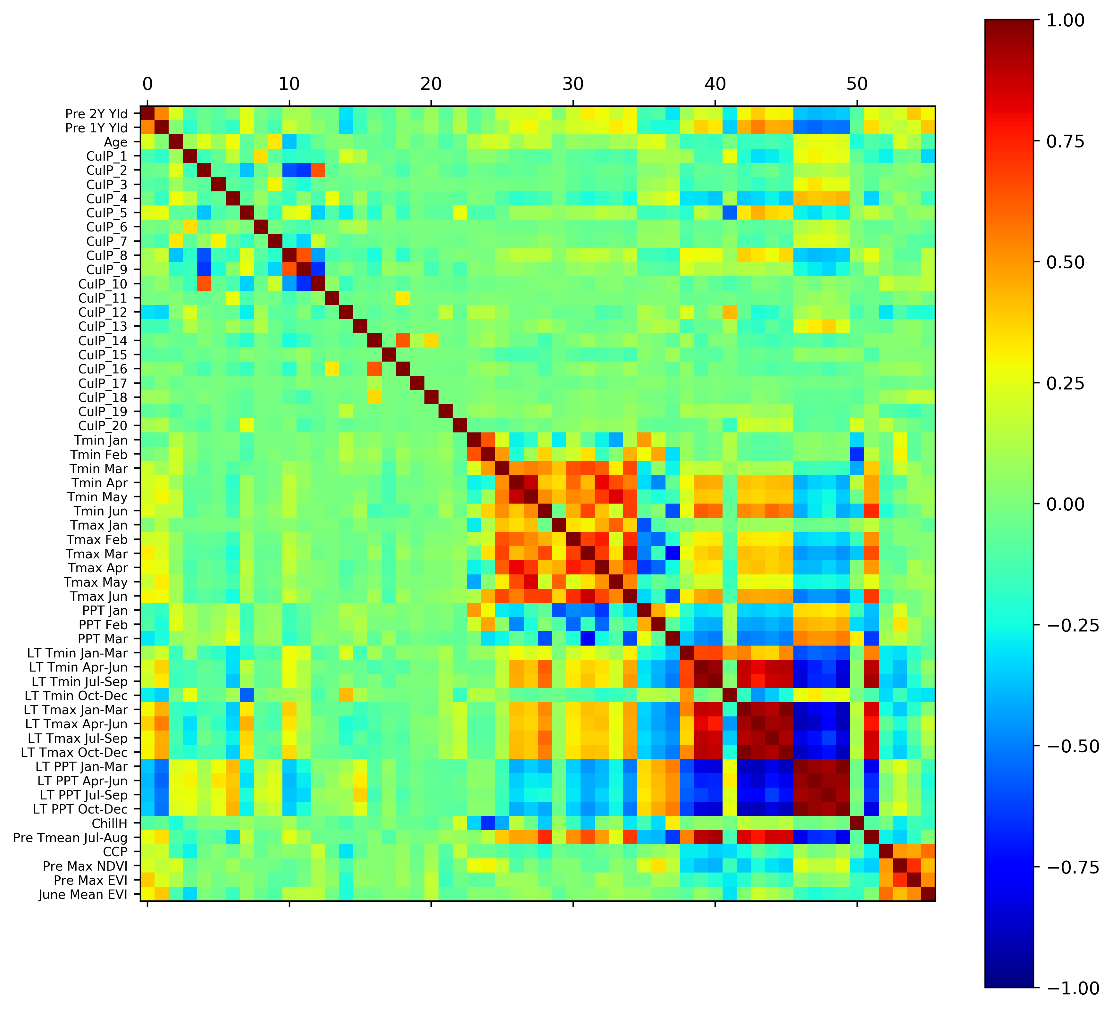


**Supplementary Figure 1.** Correlation matrix of input variables. See Table 1 for detailed variable names.

# Supplementary Table*

| **Prediction Time** | **Using Previous Years’ Yields** | **R^2^** | **RMSE (kg/ha)** | **RPIQ** |
| --- | --- | --- | --- | --- |
| Early-season | Previous two years | 0.70 (0.04) | 338 (34.4) | 2.60 (0.25) |
|  | Previous three years | 0.69 (0.03) | 344 (33.0) | 2.55 (0.22) |
|  | Previous four years | 0.68 (0.05) | 347 (37.5) | 2.55 (0.32) |
| Mid-season | Previous two years | 0.71 (0.03) | 331 (31.9) | 2.65 (0.22) |
|  | Previous three years | 0.71 (0.03) | 334 (29.1) | 2.63 (0.22) |
|  | Previous four years | 0.70 (0.03) | 341 (28.8) | 2.57 (0.19) |

**Supplementary Table 1.** Explore using different years of historical yields in yield prediction models

(*Note: the total number of available samples were reduced from 990 when using previous two years’ yields to 699 when using previous four years’ yields as predictors, since some orchards need to be excluded if they do not have that many years’ yield records. The experimental results shown in this Table were conducted based on the 699 available samples under the cross-validation scheme).
